# Dichlorido{2-morpholino-*N*-[1-(2-pyrid­yl)ethyl­idene]ethanamine-κ^3^ *N*,*N*′,*N*′′}manganese(II)

**DOI:** 10.1107/S1600536810050221

**Published:** 2010-12-04

**Authors:** Nurul Azimah Ikmal Hisham, Nura Suleiman Gwaram, Hamid Khaledi, Hapipah Mohd Ali

**Affiliations:** a Department of Chemistry, University of Malaya, 50603 Kuala Lumpur, Malaysia

## Abstract

In the title compound, [MnCl_2_(C_13_H_19_N_3_O)], the Mn^II^ ion is penta­coordinated in a distorted square-pyramidal geometry. The coordination environment is defined by the *N*,*N*′,*N*′′-tridentate Schiff base ligand and one Cl atom in the basal positions and one Cl atom in the apical position. In the crystal, inter­molecular C—H⋯Cl hydrogen bonds link the mol­ecules into a three-dimensional network. An intra­molecular C—H⋯Cl hydrogen bond is also observed.

## Related literature

For the crystal structure of the analogous Cd^II^ complex, see: Ikmal Hisham *et al.* (2010 ▶). For the crystal structure of [MnCl_2_(C_24_H_25_N_3_)], a structurally similar Mn^II^ complex, see: Schmiege *et al.* (2007 ▶).
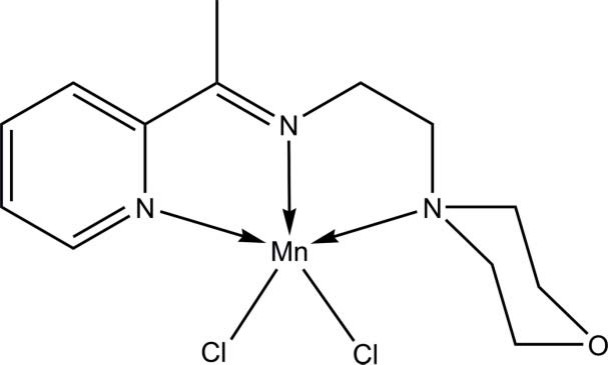


## Experimental

### 

#### Crystal data

[MnCl_2_(C_13_H_19_N_3_O)]
*M* *_r_* = 359.15
Monoclinic, 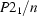

*a* = 9.6117 (6) Å
*b* = 13.8507 (8) Å
*c* = 12.1330 (7) Å
β = 106.738 (1)°
*V* = 1546.82 (16) Å^3^
*Z* = 4
Mo *K*α radiation
μ = 1.20 mm^−1^
*T* = 100 K
0.40 × 0.35 × 0.25 mm

#### Data collection

Bruker APEXII CCD diffractometer
Absorption correction: multi-scan (*SADABS*; Sheldrick, 1996 ▶) *T*_min_ = 0.646, *T*_max_ = 0.754
15613 measured reflections
3543 independent reflections
3370 reflections with *I* > 2σ(*I*)
*R*_int_ = 0.019

#### Refinement

*R*[*F* ^2^ > 2σ(*F* ^2^)] = 0.020
*wR*(*F* ^2^) = 0.053
*S* = 1.11
3543 reflections
182 parameters
H-atom parameters constrained
Δρ_max_ = 0.33 e Å^−3^
Δρ_min_ = −0.23 e Å^−3^

### 

Data collection: *APEX2* (Bruker, 2007 ▶); cell refinement: *SAINT* (Bruker, 2007 ▶); data reduction: *SAINT*; program(s) used to solve structure: *SHELXS97* (Sheldrick, 2008 ▶); program(s) used to refine structure: *SHELXL97* (Sheldrick, 2008 ▶); molecular graphics: *XP* in *SHELXTL* (Sheldrick, 2008 ▶); software used to prepare material for publication: *SHELXL97* and *publCIF* (Westrip, 2010 ▶).

## Supplementary Material

Crystal structure: contains datablocks I, global. DOI: 10.1107/S1600536810050221/is2637sup1.cif

Structure factors: contains datablocks I. DOI: 10.1107/S1600536810050221/is2637Isup2.hkl

Additional supplementary materials:  crystallographic information; 3D view; checkCIF report

## Figures and Tables

**Table 1:** Hydrogen-bond geometry (Å, °)

*D*—H⋯*A*	*D*—H	H⋯*A*	*D*⋯*A*	*D*—H⋯*A*
C4—H4⋯Cl1^i^	0.95	2.71	3.6257 (13)	163
C7—H7*C*⋯Cl2^ii^	0.98	2.75	3.6202 (13)	149
C8—H8*A*⋯Cl2^iii^	0.99	2.83	3.7207 (14)	151
C12—H12*B*⋯Cl1	0.99	2.77	3.5904 (14)	141

Symmetry codes: (i) 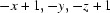; (ii) 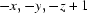; (iii) 
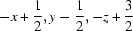
.

**Fig. 1.**
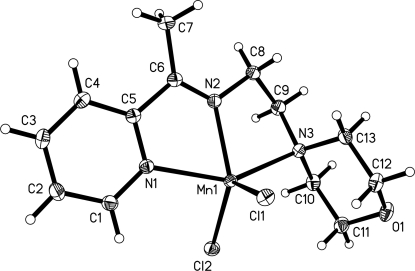
Thermal ellipsoid plot of the title compound at the 50% probability level. Hydrogen atoms are drawn as spheres of arbitrary radii.

## supplementary crystallographic information

### Comment

The title compound is isostructure to the recently reported Cd^II^ complex (Ikmal Hisham *et al.*, 2010). The Mn^II ^ion is five-coordinated by the Schiff base 2-morpholino-*N*-[1-(2-pyridyl)ethylidene]ethanamine and two Cl atoms in a distorted square-pyramidal environment (τ = 0.22). The Mn—Cl and Mn—N bond lengths in the present structure is similar to those in [MnCl_2_(C_24_H_25_N_3_)], the structurally closest Mn^II^ complex (Schmiege *et al.*, 2007). In the crystal structure, intermolecular C—H···Cl hydrogen bonds link the adjacent molecules into a three-dimensional network. An intramolecular C—H···Cl hydrogen bonding has also been observed.

### Experimental

A mixture of 2-acetylpyridine (0.2 g, 1.65 mmol) and 4-(2-aminoethyl)morpholine (0.21 g, 1.65 mmol in ethanol (20 ml) was refluxed for 2 hr followed by addition of a solution of manganese(II) chloride (0.206 g, 1.65 mmol) in a minimum amount of water. The resulting solution was refluxed for 30 min, then set aside at room temperature. The crystals of the manganese(II) complex were obtained after a few days.

### Refinement

The hydrogen atoms were placed at calculated positions (C—H 0.95–0.99 Å) and were treated as riding on their parent atoms with *U*_iso_(H) set to 1.2 or 1.5*U*_eq_(C).

### Crystal data

**Table:**

[MnCl_2_(C_13_H_19_N_3_O)]	*F*(000) = 740
*M**_r_* = 359.15	*D*_x_ = 1.542 Mg m^−^^3^
Monoclinic, *P*2_1_/*n*	Mo *K*α radiation, λ = 0.71073 Å
Hall symbol: -P 2yn	Cell parameters from 9925 reflections
*a* = 9.6117 (6) Å	θ = 2.2–31.4°
*b* = 13.8507 (8) Å	µ = 1.20 mm^−^^1^
*c* = 12.1330 (7) Å	*T* = 100 K
β = 106.738 (1)°	Block, orange
*V* = 1546.82 (16) Å^3^	0.40 × 0.35 × 0.25 mm
*Z* = 4	

### Data collection

**Table:**

Bruker APEXII CCD diffractometer	3543 independent reflections
Radiation source: fine-focus sealed tube	3370 reflections with *I* > 2σ(*I*)
graphite	*R*_int_ = 0.019
φ and ω scans	θ_max_ = 27.5°, θ_min_ = 2.3°
Absorption correction: multi-scan (*SADABS*; Sheldrick, 1996)	*h* = −12→12
*T*_min_ = 0.646, *T*_max_ = 0.754	*k* = −16→17
15613 measured reflections	*l* = −15→15

### Refinement

**Table:**

Refinement on *F*^2^	Primary atom site location: structure-invariant direct methods
Least-squares matrix: full	Secondary atom site location: difference Fourier map
*R*[*F*^2^ > 2σ(*F*^2^)] = 0.020	Hydrogen site location: inferred from neighbouring sites
*wR*(*F*^2^) = 0.053	H-atom parameters constrained
*S* = 1.11	*w* = 1/[σ^2^(*F*_o_^2^) + (0.0202*P*)^2^ + 0.8164*P*] where *P* = (*F*_o_^2^ + 2*F*_c_^2^)/3
3543 reflections	(Δ/σ)_max_ = 0.001
182 parameters	Δρ_max_ = 0.33 e Å^−^^3^
0 restraints	Δρ_min_ = −0.23 e Å^−^^3^

### Special details

**Table:**

Geometry. All e.s.d.'s (except the e.s.d. in the dihedral angle between two l.s. planes) are estimated using the full covariance matrix. The cell e.s.d.'s are taken into account individually in the estimation of e.s.d.'s in distances, angles and torsion angles; correlations between e.s.d.'s in cell parameters are only used when they are defined by crystal symmetry. An approximate (isotropic) treatment of cell e.s.d.'s is used for estimating e.s.d.'s involving l.s. planes.
Refinement. Refinement of *F*^2^ against ALL reflections. The weighted *R*-factor *wR* and goodness of fit *S* are based on *F*^2^, conventional *R*-factors *R* are based on *F*, with *F* set to zero for negative *F*^2^. The threshold expression of *F*^2^ > σ(*F*^2^) is used only for calculating *R*-factors(gt) *etc*. and is not relevant to the choice of reflections for refinement. *R*-factors based on *F*^2^ are statistically about twice as large as those based on *F*, and *R*- factors based on ALL data will be even larger.

### Fractional atomic coordinates and isotropic or equivalent isotropic displacement parameters (Å^2^)

**Table:**

	*x*	*y*	*z*	*U*_iso_*/*U*_eq_	
Mn1	0.227997 (19)	0.094201 (13)	0.662679 (15)	0.01293 (6)	
Cl1	0.46326 (3)	0.14018 (2)	0.77456 (2)	0.01695 (7)	
Cl2	0.04111 (3)	0.21023 (2)	0.63296 (3)	0.01823 (7)	
O1	0.18363 (10)	0.06033 (8)	1.01401 (8)	0.0220 (2)	
N1	0.26463 (11)	0.11770 (8)	0.48657 (9)	0.0147 (2)	
N2	0.24422 (11)	−0.04625 (8)	0.58539 (9)	0.0152 (2)	
N3	0.15480 (11)	−0.01520 (8)	0.78545 (8)	0.0144 (2)	
C1	0.25654 (14)	0.20208 (9)	0.43212 (11)	0.0176 (2)	
H1	0.2251	0.2573	0.4647	0.021*	
C2	0.29241 (14)	0.21242 (10)	0.32937 (11)	0.0195 (3)	
H2	0.2854	0.2735	0.2926	0.023*	
C3	0.33834 (14)	0.13223 (10)	0.28210 (11)	0.0198 (3)	
H3	0.3649	0.1375	0.2127	0.024*	
C4	0.34524 (13)	0.04350 (10)	0.33739 (11)	0.0172 (2)	
H4	0.3760	−0.0127	0.3061	0.021*	
C5	0.30645 (12)	0.03845 (9)	0.43907 (10)	0.0141 (2)	
C6	0.29694 (13)	−0.05409 (9)	0.50011 (10)	0.0143 (2)	
C7	0.34077 (14)	−0.14781 (9)	0.45884 (11)	0.0178 (2)	
H7A	0.3963	−0.1860	0.5249	0.027*	
H7B	0.4010	−0.1352	0.4076	0.027*	
H7C	0.2537	−0.1837	0.4169	0.027*	
C8	0.21498 (15)	−0.13062 (9)	0.64771 (11)	0.0184 (3)	
H8A	0.3055	−0.1530	0.7043	0.022*	
H8B	0.1756	−0.1841	0.5935	0.022*	
C9	0.10472 (15)	−0.09964 (9)	0.70858 (11)	0.0187 (3)	
H9A	0.0123	−0.0831	0.6503	0.022*	
H9B	0.0856	−0.1544	0.7547	0.022*	
C10	0.02951 (13)	0.01896 (10)	0.82360 (11)	0.0182 (3)	
H10A	−0.0132	−0.0362	0.8544	0.022*	
H10B	−0.0461	0.0455	0.7568	0.022*	
C11	0.07601 (15)	0.09582 (10)	0.91531 (11)	0.0209 (3)	
H11A	0.1154	0.1519	0.8835	0.025*	
H11B	−0.0096	0.1179	0.9382	0.025*	
C12	0.30849 (14)	0.03123 (10)	0.98074 (11)	0.0201 (3)	
H12A	0.3838	0.0067	1.0490	0.024*	
H12B	0.3490	0.0878	0.9507	0.024*	
C13	0.27076 (14)	−0.04662 (10)	0.88940 (11)	0.0182 (2)	
H13A	0.3589	−0.0638	0.8670	0.022*	
H13B	0.2380	−0.1051	0.9217	0.022*	

### Atomic displacement parameters (Å^2^)

**Table:**

	*U*^11^	*U*^22^	*U*^33^	*U*^12^	*U*^13^	*U*^23^
Mn1	0.01418 (10)	0.01214 (10)	0.01314 (9)	−0.00041 (7)	0.00499 (7)	−0.00097 (6)
Cl1	0.01485 (13)	0.01773 (15)	0.01853 (14)	−0.00194 (11)	0.00523 (11)	−0.00213 (11)
Cl2	0.01707 (14)	0.01822 (15)	0.01954 (14)	0.00326 (11)	0.00548 (11)	−0.00012 (11)
O1	0.0215 (5)	0.0319 (5)	0.0142 (4)	0.0012 (4)	0.0078 (4)	−0.0010 (4)
N1	0.0148 (5)	0.0144 (5)	0.0146 (5)	−0.0003 (4)	0.0039 (4)	−0.0008 (4)
N2	0.0172 (5)	0.0137 (5)	0.0144 (5)	−0.0015 (4)	0.0042 (4)	−0.0005 (4)
N3	0.0146 (5)	0.0154 (5)	0.0136 (5)	−0.0011 (4)	0.0045 (4)	−0.0010 (4)
C1	0.0190 (6)	0.0155 (6)	0.0177 (6)	0.0002 (5)	0.0043 (5)	−0.0003 (5)
C2	0.0216 (6)	0.0184 (6)	0.0172 (6)	−0.0021 (5)	0.0035 (5)	0.0030 (5)
C3	0.0205 (6)	0.0252 (7)	0.0139 (5)	−0.0032 (5)	0.0055 (5)	−0.0001 (5)
C4	0.0160 (6)	0.0193 (6)	0.0165 (6)	−0.0009 (5)	0.0049 (5)	−0.0031 (5)
C5	0.0116 (5)	0.0152 (6)	0.0141 (5)	−0.0004 (4)	0.0016 (4)	−0.0016 (4)
C6	0.0117 (5)	0.0148 (6)	0.0147 (5)	−0.0009 (4)	0.0011 (4)	−0.0023 (4)
C7	0.0185 (6)	0.0150 (6)	0.0206 (6)	0.0007 (5)	0.0067 (5)	−0.0030 (5)
C8	0.0265 (6)	0.0123 (6)	0.0172 (6)	−0.0025 (5)	0.0078 (5)	−0.0002 (5)
C9	0.0222 (6)	0.0161 (6)	0.0184 (6)	−0.0064 (5)	0.0068 (5)	−0.0016 (5)
C10	0.0143 (6)	0.0237 (7)	0.0179 (6)	−0.0007 (5)	0.0067 (5)	0.0016 (5)
C11	0.0215 (6)	0.0248 (7)	0.0185 (6)	0.0045 (5)	0.0090 (5)	0.0002 (5)
C12	0.0177 (6)	0.0277 (7)	0.0151 (6)	−0.0004 (5)	0.0050 (5)	−0.0006 (5)
C13	0.0185 (6)	0.0195 (6)	0.0159 (6)	0.0022 (5)	0.0039 (5)	0.0030 (5)

### Geometric parameters (Å, °)

**Table:**

Mn1—N2	2.1845 (11)	C4—H4	0.9500
Mn1—N1	2.2867 (10)	C5—C6	1.4963 (17)
Mn1—Cl2	2.3593 (4)	C6—C7	1.4949 (17)
Mn1—Cl1	2.3651 (4)	C7—H7A	0.9800
Mn1—N3	2.3694 (10)	C7—H7B	0.9800
O1—C11	1.4255 (16)	C7—H7C	0.9800
O1—C12	1.4300 (15)	C8—C9	1.5178 (18)
N1—C1	1.3337 (17)	C8—H8A	0.9900
N1—C5	1.3535 (16)	C8—H8B	0.9900
N2—C6	1.2811 (16)	C9—H9A	0.9900
N2—C8	1.4624 (16)	C9—H9B	0.9900
N3—C10	1.4864 (15)	C10—C11	1.5116 (18)
N3—C9	1.4869 (16)	C10—H10A	0.9900
N3—C13	1.4875 (16)	C10—H10B	0.9900
C1—C2	1.3932 (17)	C11—H11A	0.9900
C1—H1	0.9500	C11—H11B	0.9900
C2—C3	1.3796 (19)	C12—C13	1.5135 (18)
C2—H2	0.9500	C12—H12A	0.9900
C3—C4	1.3925 (19)	C12—H12B	0.9900
C3—H3	0.9500	C13—H13A	0.9900
C4—C5	1.3896 (17)	C13—H13B	0.9900
			
N2—Mn1—N1	71.12 (4)	C6—C7—H7B	109.5
N2—Mn1—Cl2	133.34 (3)	H7A—C7—H7B	109.5
N1—Mn1—Cl2	94.33 (3)	C6—C7—H7C	109.5
N2—Mn1—Cl1	108.15 (3)	H7A—C7—H7C	109.5
N1—Mn1—Cl1	96.83 (3)	H7B—C7—H7C	109.5
Cl2—Mn1—Cl1	117.667 (13)	N2—C8—C9	106.90 (10)
N2—Mn1—N3	76.77 (4)	N2—C8—H8A	110.3
N1—Mn1—N3	146.31 (4)	C9—C8—H8A	110.3
Cl2—Mn1—N3	100.42 (3)	N2—C8—H8B	110.3
Cl1—Mn1—N3	102.67 (3)	C9—C8—H8B	110.3
C11—O1—C12	108.96 (9)	H8A—C8—H8B	108.6
C1—N1—C5	118.81 (11)	N3—C9—C8	112.48 (10)
C1—N1—Mn1	125.80 (8)	N3—C9—H9A	109.1
C5—N1—Mn1	115.27 (8)	C8—C9—H9A	109.1
C6—N2—C8	121.97 (11)	N3—C9—H9B	109.1
C6—N2—Mn1	121.06 (9)	C8—C9—H9B	109.1
C8—N2—Mn1	116.20 (8)	H9A—C9—H9B	107.8
C10—N3—C9	107.50 (10)	N3—C10—C11	111.04 (10)
C10—N3—C13	107.79 (9)	N3—C10—H10A	109.4
C9—N3—C13	109.27 (10)	C11—C10—H10A	109.4
C10—N3—Mn1	113.96 (8)	N3—C10—H10B	109.4
C9—N3—Mn1	102.11 (7)	C11—C10—H10B	109.4
C13—N3—Mn1	115.75 (7)	H10A—C10—H10B	108.0
N1—C1—C2	122.63 (12)	O1—C11—C10	111.39 (11)
N1—C1—H1	118.7	O1—C11—H11A	109.3
C2—C1—H1	118.7	C10—C11—H11A	109.3
C3—C2—C1	118.72 (12)	O1—C11—H11B	109.3
C3—C2—H2	120.6	C10—C11—H11B	109.3
C1—C2—H2	120.6	H11A—C11—H11B	108.0
C2—C3—C4	119.13 (12)	O1—C12—C13	111.34 (11)
C2—C3—H3	120.4	O1—C12—H12A	109.4
C4—C3—H3	120.4	C13—C12—H12A	109.4
C5—C4—C3	118.98 (12)	O1—C12—H12B	109.4
C5—C4—H4	120.5	C13—C12—H12B	109.4
C3—C4—H4	120.5	H12A—C12—H12B	108.0
N1—C5—C4	121.71 (11)	N3—C13—C12	112.05 (11)
N1—C5—C6	114.59 (10)	N3—C13—H13A	109.2
C4—C5—C6	123.57 (11)	C12—C13—H13A	109.2
N2—C6—C7	124.01 (12)	N3—C13—H13B	109.2
N2—C6—C5	114.95 (11)	C12—C13—H13B	109.2
C7—C6—C5	120.98 (11)	H13A—C13—H13B	107.9
C6—C7—H7A	109.5		

### Hydrogen-bond geometry (Å, °)

**Table:**

*D*—H···*A*	*D*—H	H···*A*	*D*···*A*	*D*—H···*A*
C4—H4···Cl1^i^	0.95	2.71	3.6257 (13)	163.
C7—H7C···Cl2^ii^	0.98	2.75	3.6202 (13)	149.
C8—H8A···Cl2^iii^	0.99	2.83	3.7207 (14)	151.
C12—H12B···Cl1	0.99	2.77	3.5904 (14)	141.

Symmetry codes: (i) −*x*+1, −*y*, −*z*+1; (ii) −*x*, −*y*, −*z*+1; (iii) −*x*+1/2, *y*−1/2, −*z*+3/2.
